# Global dissection of alternative splicing uncovers transcriptional diversity in tissues and associates with the flavonoid pathway in tea plant (*Camellia sinensis*)

**DOI:** 10.1186/s12870-018-1497-9

**Published:** 2018-11-06

**Authors:** Junyan Zhu, Xuewen Wang, Qingshan Xu, Shiqi Zhao, Yuling Tai, Chaoling Wei

**Affiliations:** 1 State Key Laboratory of Tea Plant Biology and Utilization/Key Laboratory of Tea Biology and Processing, Ministry of Agriculture, Anhui Agricultural University, West 130 Changjiang Road, Hefei, 230036 Anhui People’s Republic of China; 2 Department of Genetics, University of Georgia, 120 E Green Street, Athens, GA 30602 USA

**Keywords:** Alternative splicing, *Camellia sinensis*, Tissue-specificity, Flavonoid

## Abstract

**Background:**

Alternative splicing (AS) regulates mRNA at the post-transcriptional level to change gene function in organisms. However, little is known about the AS and its roles in tea plant (*Camellia sinensis*), widely cultivated for making a popular beverage tea.

**Results:**

In our study, the AS landscape and dynamics were characterized in eight tissues (bud, young leaf, summer mature leaf, winter old leaf, stem, root, flower, fruit) of tea plant by Illumina RNA-Seq and confirmed by Iso-Seq. The most abundant AS (~ 20%) was intron retention and involved in RNA processes. The some alternative splicings were found to be tissue specific in stem and root etc. Thirteen co-expressed modules of AS transcripts were identified, which revealed a similar pattern between the bud and young leaves as well as a distinct pattern between seasons. AS events of structural genes including anthocyanidin reductase and MYB transcription factors were involved in biosynthesis of flavonoid, especially in vegetative tissues. The AS isoforms rather than the full-length ones were the major transcripts involved in flavonoid synthesis pathway, and is positively correlated with the catechins content conferring the tea taste. We propose that the AS is an important functional mechanism in regulating flavonoid metabolites.

**Conclusion:**

Our study provides the insight into the AS events underlying tea plant’s uniquely different developmental process and highlights the important contribution and efficacy of alternative splicing regulatory function to biosynthesis of flavonoids.

**Electronic supplementary material:**

The online version of this article (10.1186/s12870-018-1497-9) contains supplementary material, which is available to authorized users.

## Background

The perennial tea plant (*Camellia sinensis*) is an important economic woody crop, and is cultivated from tropical to temperate regions [1]. The leaves of the plant are used for making tea beverage, which is popular due to good taste, attractive aroma and health-promoting effects [2]. Flavonoids are major secondary metabolites present in tea plant which closely relate to the rich flavors of tea infusions [3]. The levels of secondary metabolites in different tissues of tea plant change with different developmental stages. These changes were reported to be regulated by the expression of mRNA transcripts [1, 4, 5].

Alternative splicing (AS) is alternative splice site selection, which results in the generation of multiple isoforms from precursor-mRNA (pre-mRNA) transcripts [6, 7]; these AS transcripts are universally present in plants and animals [8]. AS greatly contributes to transcriptome and proteome diversities, and may result in differences in protein stability, sub-cellular localization or function [7, 9]. In plants, increasing numbers of studies have reported large numbers of genes that undergo extensive AS under environmental stresses and during plant developmental processes, such as heat stress, flowering timing and the circadian clock [10–14]. Furthermore, analyses of comprehensive transcriptomes from different tissues indicate that almost every intron-containing gene generates multiple tissue-specific splice variants [15, 16]. In Arabidopsis, an abnormally expressed flower-specific YUCCA4 transcript isoform could encode a protein that is localized to the endoplasmic reticulum (ER), differing from the cytosol localization of its ubiquitously expressed transcript [17]. In addition, alternative splicing of zinc-induced facilitator-like 1 (ZIFL1) yields two isoforms; ZIFL1.1 influences cellular auxin efflux and polar auxin transport in roots, whereas ZIFL1.3 regulates stomatal movement. Moreover, AS events are involved in secondary metabolite biosynthesis pathways during plant development, such as phenolic acid, phylloquinone, salicylic acid and shikimate biosynthesis pathways [18–20]. Isochorismate synthase (ICS) is an enzyme necessary for salicylic acid (SA) biosynthesis, and the emergence of AS isoforms affects its activity in vivo; splice variants of ICS could not complement the function of the full length enzyme in transgenic Arabidopsis [19]. Despite some studies having explored AS-mediated regulation of flavonoid biosynthesis [21, 22], very little is known of how and by which isoforms modulation occurs in the expression of related structural genes and transcription factors in the flavonoid biosynthesis pathway.

This study used genome-wide analysis of Illumina and SMRT RNA-Seq data from eight developing tissues, and we observed that a large number of genes were alternatively spliced in tea plant. Furthermore, a number of tissue-specific AS genes were identified by WGCNA analysis, and these may play important roles in tissue development. Many AS events were observed in flavonoid biosynthesis pathway gene transcripts, and the majority were highly abundant in vegetative tissues. Unexpectedly, the expression analysis indicated some flavonoid-derived AS transcripts were predominantly expressed in leaf relative to their full-length transcripts; also, their expression levels were highly correlated with the accumulation of catechins, suggesting these AS transcripts may function as major transcripts instead of their full-length counterparts. Together, these results highlight the underlying alternative splicing based regulatory mechanisms in tea plant development, and improve our understanding of the diverse functions of AS.

## Results

### Identification and classification of AS events

**Fig. 1:**
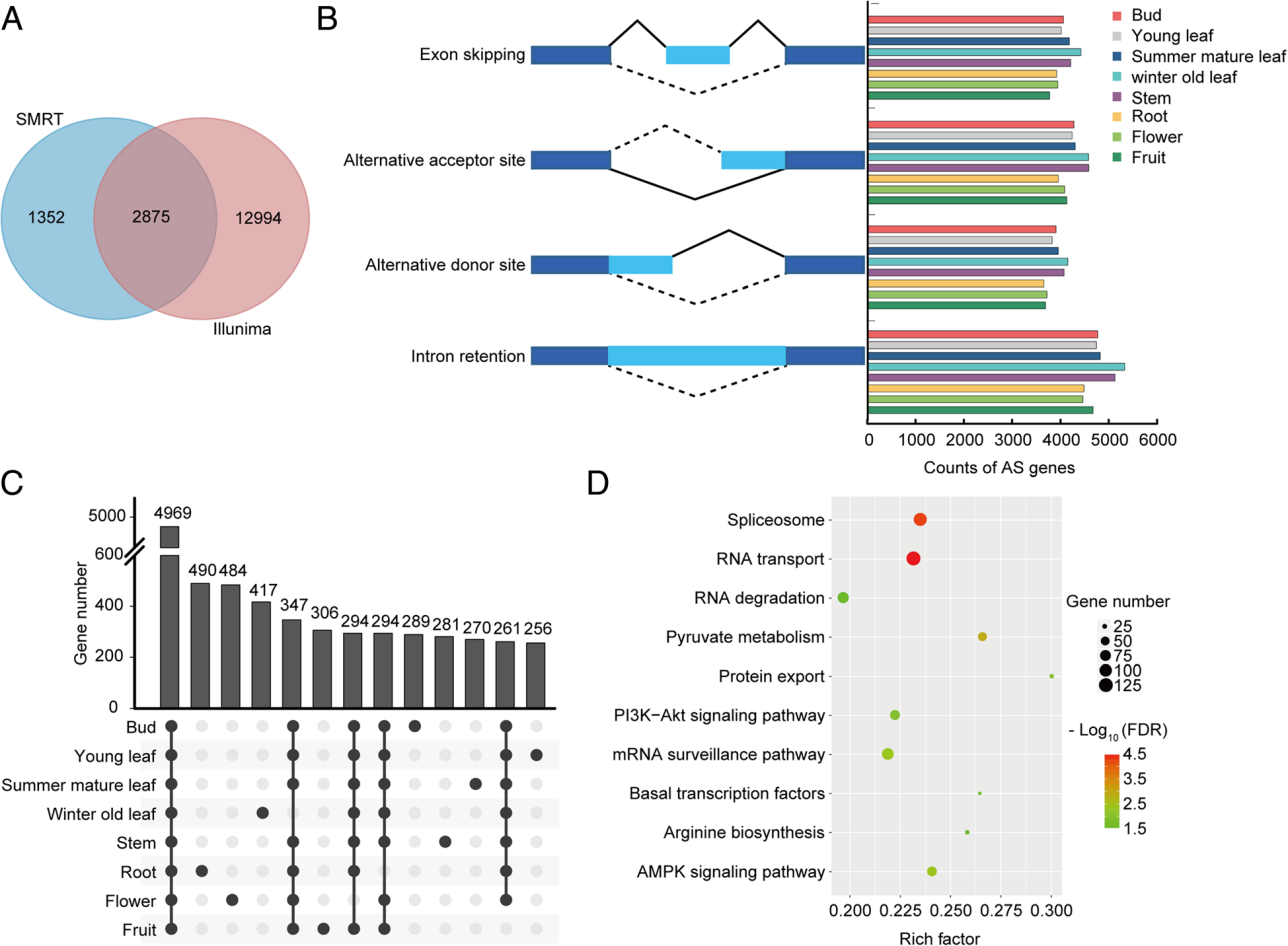
Comparative analysis of different AS isoforms among eight tissues of tea plant. **a** Venn diagram showing the common and unique AS genes determined with Illumina and SMRT sequencing. **b** Distribution of different types of alternative splicing events in eight tissues. **c** Overlap of AS genes among eight tissues. **d** KEGG pathway enrichment for AS genes from eight tissues. The area of each colored circle is proportional to the size scale of genes involved in each pathway. The color indicates the q-value, and the x-axis is the Rich Factor

To identify AS events in the transcriptome, we generated 522,811,493 clean Illumina RNA-seq reads and 80,217 SMRT subreads from eight tissues of tea plant. In our study, a total of 69,569 AS events were identified in 15,869 AS genes, which accounted for 46.8% of the total expressed genes from eight samples (Table 1). An additional 9503 AS events in 4227 genes were identified from 136 Mb SMRT Iso-Seq reads, which were generated from the same eight tissue-derived samples. Among these AS, 2875 AS genes were present in both sequencing data sets (Fig. 1a). Furthermore, approximately 81.9% of AS transcripts were uniquely identified by Illumina sequencing;the lower number of AS transcripts detected from SMRT data may have resulted from the low coverage of the SMRT sequencing reads generated here. Therefore, we focused on the AS analysis detected in Illumina data set and used the SMRT sequencing data to verify some of the AS isoforms.

**Table 1:** Statistics of different AS events obtained from the Illumina and SMRT libraries generated in this study

	Illumina		SMRT	
	Number	Percentage	Number	Percentage
Alternative donor site	9752	14.01%	836	8.79%
Alternative acceptor site	10,850	15.59%	1404	14.77%
Intron retention	14,140	20.32%	3079	32.40%
Exon skipping	10,477	15.05%	626	6.58%
Others	24,350	35.03%	3558	37.46%
Total events	69,569	100.00%	9503	100.00%

‘Illumina’ indicates the Illumina paired-end RNA-seq data generated from the eight tea plant tissues. ‘SMRT’ indicates the SMRT sequencing data generated from the eight pools of tea plant tissues. “Percentage” indicates the ratio of each AS type to the total events for each library

Among the 69,569 AS events, 20.32% resulted from intron retention (IR), followed by alternative acceptor sites (AA, 15.59%), exon skipping (ES, 15.05%), and alternative donor sites (AD, 14.01%) (Table 1). A similar pattern of AS abundance was observed in each tissue (Fig. 1b, Additional file 1: Table S1). These patterns were in accordance with previous observations in *Arabidopsis thaliana*, *Brachypodium distachyon* and *Glycine max* [23–25]. In addition, a large proportion of “other” AS types were detected in all samples, which may result from multiple splicing modes occurring on a single transcript. For example, the CUFF.13109.2 transcript was classified into“other” type due to the combination of AD and IR in the bud.

Among 15,869 AS genes, 4969 genes were collectively identified in eight tissues via comparisons of transcript isoforms, and a small proportion of AS genes were detected within individual tissue (Fig. 1c). To understand the biological processes in which AS genes might be involved, all the AS genes were analyzed with KEGG enrichment by bi-directional best hit method, and were highly enriched in processes related to RNA, such as RNA transport, spliceosome, RNA degradation (Fig. 1d). Similar biological processes were enriched among the individual tissues (data not shown).In addition, multiple splicing factors were identified from these conserved AS genes, such as eight serine/arginine-rich (SR), five U2af-like and three 3B-likeprotein genes (Additional file 1: Table S2).

### Co-expression network identified across tissues

**Fig. 2:**
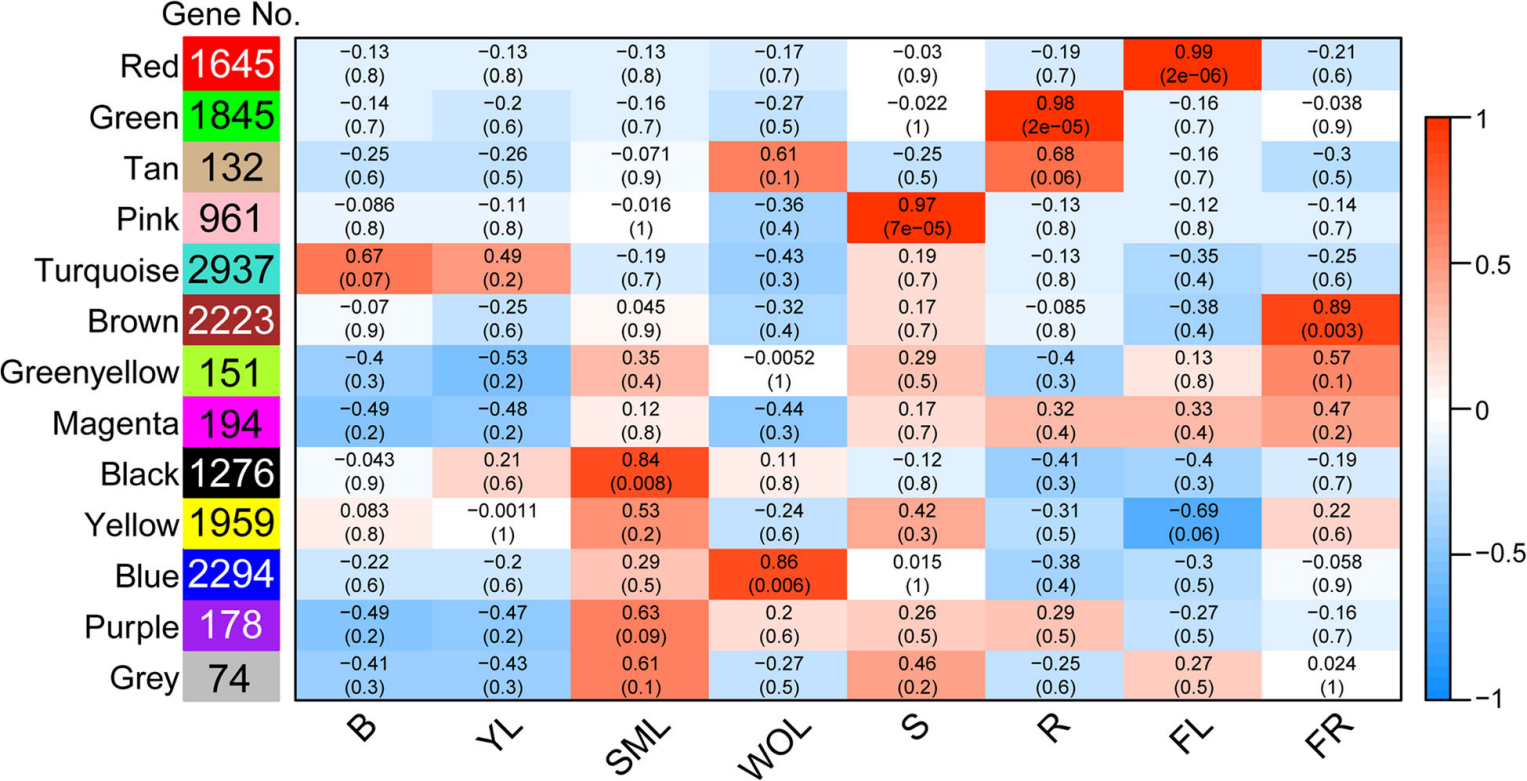
Co-expression network analysis. WGCNA analysis of AS genes in eight tissues. Each column corresponds to a specific tissue (B, bud; YL, young leaf; SML, summer mature leaf; WOL, winter old leaf; S, stem; R, root; FL, flower; FR, fruit). Each row corresponds to a module. The number of genes in each module is indicated on the left box. The color and number of each cell at the row-column intersection indicates the correlation coefficient between the module and the tissue type. A high degree of correlation between a specific module and the tissue type is indicated by dark red

**Fig. 3:**
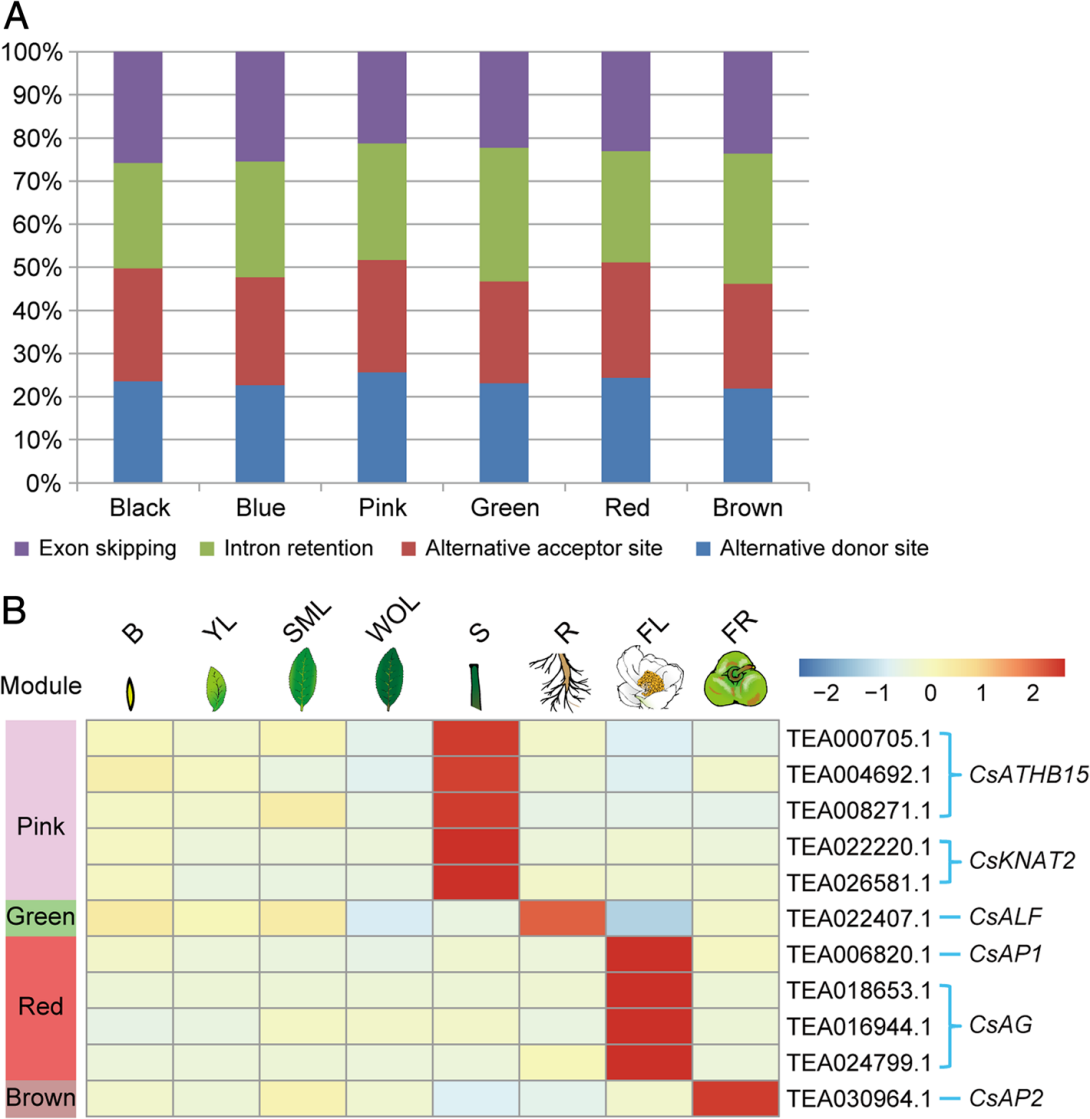
Characteristic analysis of tissue-specific AS genes. **a** Distribution of different types of alternative splicing events in eight tissues. **b** Heat map showing the transcript level in FPKM of each tissue-specific AS gene from the pink (stem) module, green (root) module, red (flower) module and brown (fruit), respectively. Red and blue colors represent high and low levels of transcript abundance, respectively

A co-expression network across eight tissues of tea plant was constructed with WGCNA (v1.29) based on pairwise correlation between common AS expression patterns [26]. Thirteen distinct modules were observed (Additional file 1: Figure S1), and the genes within these modules were partially correlated with distinct tissue types (Fig. 2). Notably, AS genes in six co-expression modules (black, blue, pink, green, red and brown) are highly expressed in a single tissue type (*r* > 0.8, *P* < 10^− 3^); hence, these six modules could be regarded as tissue specific clusters of genes. For example, the red module contains 1645 AS genes identified as FL-specific (flower), and the brown module contains 2223 FR-specific (fruit) AS genes.The splicing mode was not uniform across the six modules. The predominant AS type IR was only the most prevalent in root and fruit modules, but remained common in the other modules (Fig. 3a, Additional file 1: Table S3).

It’s remarkable that some AS genes within the turquoise module were co-expressed in the bud and the young leaf, and were enriched in the process of cell growth and death by KEGG enrichment analysis (data not shown). In addition, two structural genes involved in the flavonoid pathway with significant co-expression within the turquoise module are leucoanthocyanidin reductase (TEA027557.1) and anthocyanidin reductase (TEA022952.1). Conjecturally, as these AS genes are co-expressed in young leaf and bud, they are likely biologically relevant and their correlation may underscore the developmental relationship and molecular similarities between these two tissues. Notably, summer mature leaf (SML) and winter old leaf (WOL) are clearly divided into two distinct modules (black and blue), and no credible co-expression relationship was observed between the two tissues. Therefore, to certain degrees, the identification of SML-specific (black module) and WOL-specific (blue module) modules (Fig 3a) probably suggested that the developing divergence between AS expression patterns in summer mature leaf and winter old leaf.

### Identification of tissue-specific AS gene expression

Characterization of tissue or cell-specific genes provides a foundation for unraveling their molecular mechanisms during tissue development. We further analyzed the expression patterns of other tissue-specific AS genes. Several key regulatory genes were identified and found to be highly expressed. Three class III *HD-ZIP* genes (*ATHB15*) and three *KNOX1* transcription factors (*KNAT2*) were identified in the stem-specific module (Fig. 3), which were reported to be directly associated with vascular development and maintenance of meristematic identity in *Arabidopsis* [27]*.* The occurrences of AS events with these genes suggest that they could perform a redundant function during stem development. In Arabidopsis, the nuclear protein, *ALF* (*ABERRANT LATERAL ROOT FORMATION*) is required to maintain xylem pole pericycle cells in lateral root formation [28]. A paralog to *ALF* was found within the root-specific module, and had similar root-specific expression profiles. The ABCE classes of genes are the best characterized flower developmental genes, and their expression patterns were highly stereotypic in flower [29]. Not surprisingly, we found one *APETALA1* (*AP1*) and three AGAMOUS (*AG*) splice variants which were highly expressed in the flower-specific module. Interestingly, *AP1* and *AG* gene were detected in the fruit-specific module, but none of the A, B, C and E genes were expressed in other modules. This conservation of AS patterns in the ABCE model genes between flower and fruit suggest similar regulation in these two reproductive tissues.

### Detection and characterization of AS in genes involved in the flavonoid pathway

**Fig. 4:**
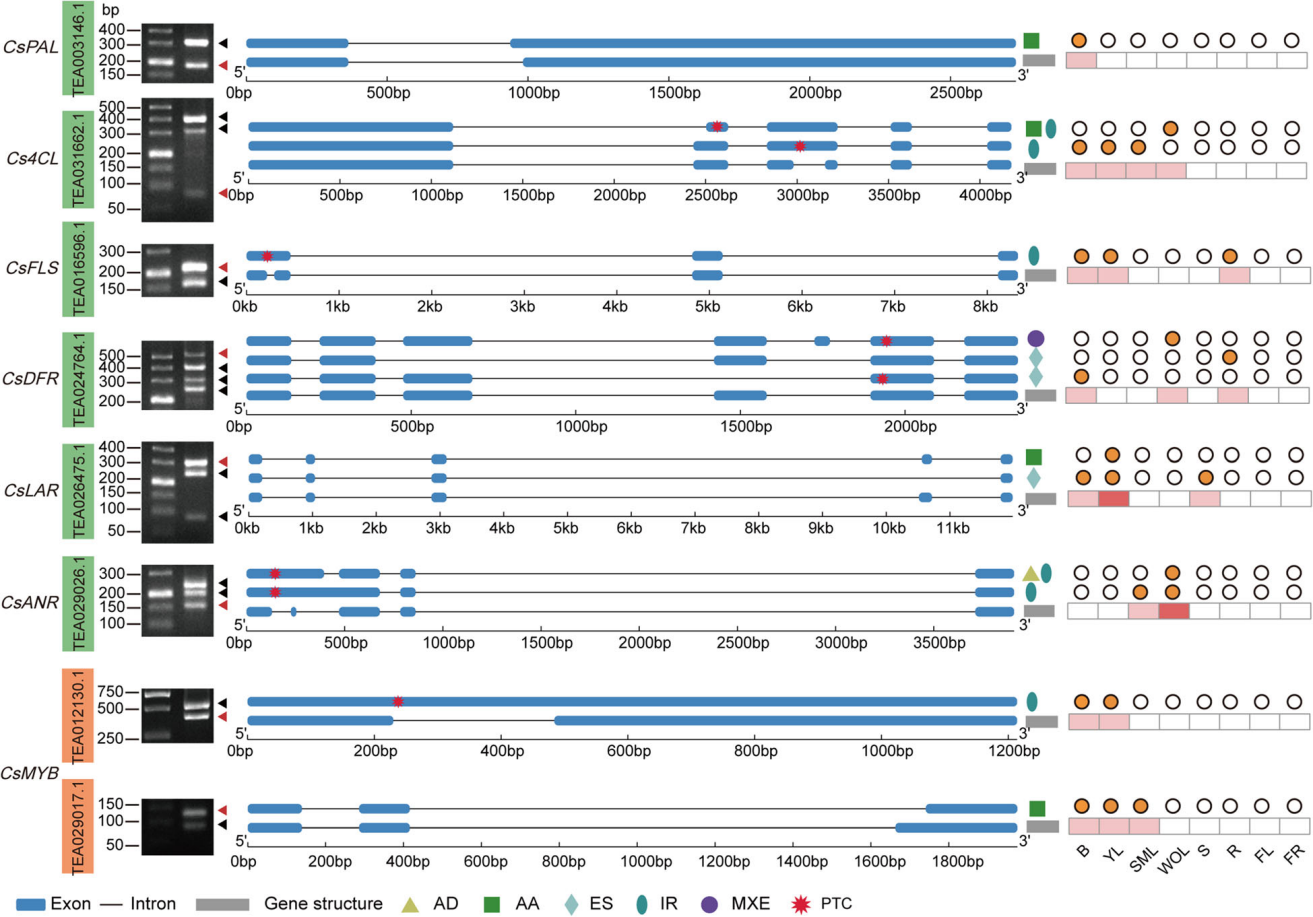
Alternatively spliced isoforms of flavonoid-related genes in tea plant. The red asterisk indicates the position of PTC. The full-length and AS isoforms on the gel images are denoted with red and black triangles, respectively. The orange solid dot indicates the targeted AS transcripts were identified in specific tissues (B, bud; YL, young leaf; SML, summer mature leaf; WOL, winter old leaf; S, stem; R, root; FL, flower; FR, fruit)

To better understand the putative influence of alternative splicing on secondary metabolism, we investigated AS transcripts of genes which are directly or indirectly involved in characteristic secondary metabolism pathways including the flavonoid, theanine and caffeine (Additional file 1: Figures S2, S3 and S4) [2, 3, 30, 31]. Interestingly, nearly all the structural flavonoid pathway genes generated multiple splicing variants, and some transcripts were tissue-specific (Additional file 1: Table S4). For instance, three different AS isoforms were produced for dihydroflavonol 4-reductase (TEA024764.1), which performs a vital rate-limiting function; these were produced in three different tissues (bud, young leaf and stem). Notably, the occurrence of flavonoid biosynthesis related AS were frequently observed in vegetative organs such as bud and leaf (Fig. 4). Furthermore, an AS type called mutually exclusive exon (MXE) was found in some transcripts, which were classified into the “other” category in the previous transcriptome analysis. To exclude the possibility that these transcripts were experimental or informatic artefacts, RT-PCR was performed for validation using primers flanking the AS site (Fig. 4).

The MYB transcription factors are key regulators of the synthesis of flavonoid-derived compounds [32]. We identified 12 homologs of flavonoid-related MYB genes in tea plant and constructed a phylogenetic tree (Additional file 1: Figure S5). Meanwhile, among these *CsMYBs*, four were regulated by AS, which may serve as an activator to mediate down-stream genes. Similarly, AS of two *CsMYBs* (TEA012130.1 and TEA029017.1) was detected in the Illumina data set and verified by RT-PCR (Fig. 4). Additionally, considering of possibility of unstable AS transcripts, five key flavonoid-related AS isoforms were selected to validate by RT-PCR, using independent samples (bud, young leaf, root and flower). The results showed that the five isoforms were detected, although the expression levels varied with different tissues (Additional file 1: Figure S6).

Sequence analysis unexpectedly revealed that most of the detected AS events resulted in introduction of a premature stop codon (PTC). Therefore they could be degraded through the nonsense-mediated decay (NMD) mechanism (Fig. 4) [33]. However, the non-PTC AS transcripts could be translated into truncated proteins despite gaining or losing parts of conserved domains, which could also lead to variants with functional differences.

### Expression of flavonoid-related AS genes was correlated with catechins

**Fig. 5:**
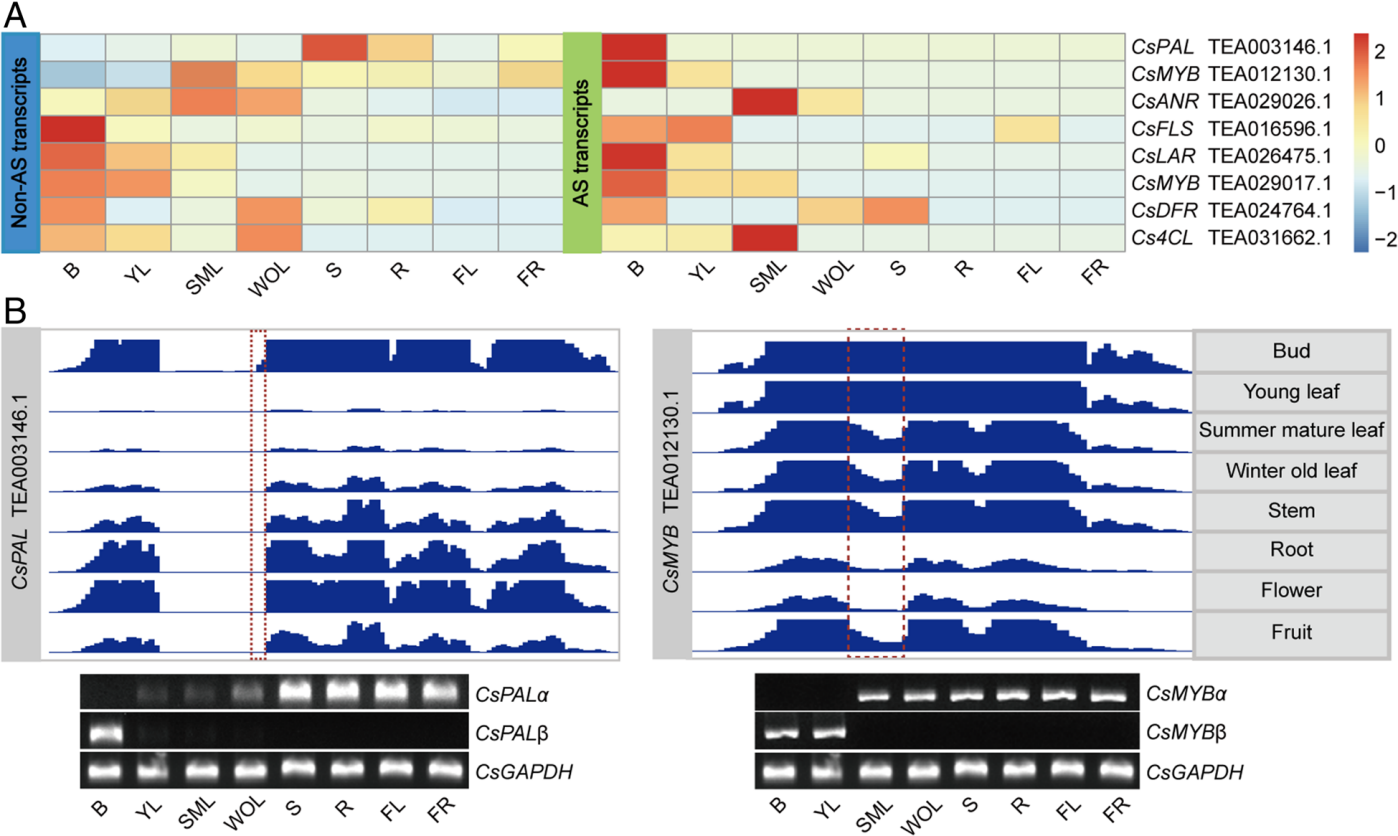
Expression patterns of flavonoid-related AS transcripts. **a** Heat map showing the FPKM of non-AS and AS transcripts in eight tissues (B, bud; YL, young leaf; SML, summer mature leaf; WOL, winter old leaf; S, stem; R, root; FL, flower; FR, fruit). Red and blue colors represent high and low transcript abundance, respectively. **b** Read coverage graphs of TEA003146.1 and TEA012130.1 derived from eight tissues viewed in IGV and validated by semi-quantitative RT-PCR. The red dotted boxes indicate the positions of alternative splicing. The RT-PCR bands are shown in the gel images beneath the graphs. The *CsGAPDH* gene was used as the internal control

To explore the extent and conservation of AS among flavonoid-related genes, we evaluated the expression patterns of these genes in different tissues using RNA-Seq data. Among eight flavonoid-related genes, six full-length transcripts were highly expressed in vegetative tissues, while the corresponding AS transcripts also followed similar expression patterns (Fig. 5a). The AS transcripts of two genes, *CsPAL* (TEA003146.1) and *CsMYB* (TEA012130.1), were the predominant isoforms in vegetative organs while their full-length transcripts exhibited completely opposite expression patterns, suggesting a potential functional divergence between these two transcripts. Furthermore, Sanger sequencing, IGV visualization and semi-quantitative RT-PCR confirmed the expression patterns of predicted alternative splicing events in the *CsPAL* and CsMYB transcripts (Fig. 5b).

To further explore the relationship between transcript expression of flavonoid-related genes and metabolites, a pearson’s correlation analysis between the expression levels in FPKM of the AS genes and total catechins’ contents indicated significant correlations (*P* < 0.05) between total catechins and *CsFLSα* (TEA016596.1), *CsLARα* (TEA026475.1), *CsMYBα* (TEA029017.1), *CsLARβ* (TEA026475.1), *CsMYBβ* (TEA012130.1) and *CsMYBβ* (TEA029017.1) (Table 2). Surprisingly, *CsANRα* (TEA029026.1) and β were not correlated with total catechins, but were significantly correlated (*P* < 0.05) with epigallocatechin (EGC). In addition, we observed high correlation (*P* < 0.01) of epicatechin-3-gallate (ECG) with AS transcripts of *CsMYBβ* (TEA012130.1) and *CsPALβ* (TEA003146.1), but no correlation with the full-length transcripts.

**Table 2:** Correlation analysis between flavonoid-related transcripts and catechins

Pearson Correlation								
		2,3-*trans*-catechin		2,3-cis-catechin				
Annotation	Gene ID	C	GC	EC	EGC	ECG	EGCG	Total catechins
	Non-AS transcripts							
CsPALα	TEA003146.1	0	0.05	− 0.25	− 0.25	− 0.53	− 0.57	− 0.58
Cs4CLα	TEA031662.1	− 0.16	− 0.2	0.05	0.26	0.49	0.80*	0.69
CsFLSα	TEA016596.1	0.3	−0.03	0.2	0.04	0.92**	0.77*	0.71*
CsDFRα	TEA024764.1	−0.09	− 0.25	− 0.22	0.03	0.46	0.44	0.35
CsLARα	TEA026475.1	0.26	0.25	0.51	0.43	0.84**	0.92**	0.93**
CsANRα	TEA029026.1	−0.09	0.53	0.32	0.81*	−0.32	0	0.2
CsMYBα	TEA012130.1	0.04	0.36	0.16	0.44	−0.62	−0.39	−0.25
CsMYBα	TEA029017.1	0.17	0.09	0.42	0.29	0.81*	0.93**	0.89**
	AS transcripts							
CsPALβ	TEA003146.1	0.41	0.08	0.27	0.08	0.87**	0.71*	0.69
Cs4CLβ	TEA031662.1	0.14	0.74*	0.65	0.91**	0.06	0.3	0.53
CsFLSβ	TEA016596.1	−0.03	−0.21	0.16	−0.04	0.84**	0.78*	0.68
CsDFRβ	TEA024764.1	0.14	0.02	−0.02	0.04	0.22	0.35	0.3
CsLARβ	TEA026475.1	0.32	0.06	0.32	0.11	0.87**	0.85**	0.79*
CsANRβ	TEA029026.1	−0.02	0.63	0.41	0.86**	−0.29	− 0.01	0.22
CsMYBβ	TEA012130.1	0.31	0.03	0.32	0.12	0.92**	0.87**	0.82*
CsMYBβ	TEA029017.1	0.3	0.38	0.58	0.56	0.78*	0.87**	0.93**

The numbers indicate the Pearson correlation coefficient. The asterisks indicate the significance level (*P < 0.05, **P < 0.01) based on a Tukey’s honestly significant difference test

## Discussion

Alternative splicing (AS) is known as an alternative expressional regulatory mechanism, which commonly occurs in plants to control gene expression, and potentially results in modified protein function [6, 7]. Little is known about underlying AS events in tea plant (*Camellia sinensis*). Although Illumina based RNA-Seq transcriptome in different tissues of tea plant had been reported [4, 34], the crucial and more flexible regulatory roles of AS were never addressed. There have been limitations considering that many previous analyses were performed without genome data and with a low depth of sequencing data; AS events in SMRT RNA-Seq data had been proposed in three secondary metabolism pathways [18]. In this study, we investigated the total AS events in different tissues of tea plant by combining Illumina and SMRT RNA-Seq technologies. AS events of key tissue-specific and flavonoid-derived transcripts were observed, and they could play important roles in producing characteristic tea flavor and for regulation of developmental processes. Thus, this study provides novel understanding of the roles of alternative splicing in tea plant.

Among the 15,869 detected genes alternatively spliced in eight tissues, the characterization of AS splicing modes and KEGG enrichment analysis were similar (Fig. 1b, d), indicating the high conservation of AS dynamics during tea plant development; A similar phenomenon was observed in soybean [25]. In contrast, we found that approximately 31.3% of AS genes to be tissue conserved, which was a greater percentage than in soybean [25]. Among these conserved AS genes, three representative types of splicing factors were found, such as eight SR genes (Additional file 1: Table S2). Previous analyses indicated that the SR protein family plays an important role in regulating AS in a tissue-specific and stress-responsive manner [35, 36]. The AS of these highly conserved splicing factors among all tissues implied that they may act as vital regulatory genes in not only self-regulation by AS, but also in regulating AS profiles of other genes during tea plant developmental stages.

Tissue specific AS may have potential functions. Studies in multiple plants have indicated that each organ or tissue has its specific AS transcripts [37, 38], which have been proven to mediate tissue differentiation and promote specialized characteristics; for example, tissue specific AS events affect polyploidy in endosperm and microgametogenesis in pollen [39–41]. In our study, although AS dynamics were conserved between leaves of different developmental stages, a set of AS genes with considerable overlap between bud and young leaf was observed, while large differences were found between SML-specific and WOL-specific AS genes (Fig. 2). This may imply that molecular features are similar between bud and young leaf, whereas SML and WOL have distinct molecular characteristics. Moreover, this speculation was strengthened by previous studies on comparative transcriptome analysis of tea leaf at different developmental stages, which examined expression of full-length transcripts [5, 42]. In addition, multiple tissue-specific AS transcripts were detected by WGCNA analysis, such as those of stem-specific *ATHB15* and flower-specific ABCE genes. A tissue-specific non-functional isoform of cyclin in maize played a role in endoreduplication [43]. Thus, the occurrence of tissue specific AS in tea plant could perform various functions during development. Our future investigations will aim to test the potential functions of tissue-specific AS.

The roles of flavonoids in plants include responses to environmental challenges, and flavonoids are contribute to the quality of tea leaves [44, 45]. Great advances had been made in understanding transcriptional regulation of the flavonoid biosynthesis pathway in tea plant, which include the characterization of structural genes and transcription factors. However, the influence of alternative splicing to flavonoid biosynthesis has not been explored. In this study, abundant AS events of structural genes and *MYB* transcription factors were characterized in the flavonoid biosynthesis pathway (Fig. 4). Interestingly, except for up-regulation of these full-length transcripts during different stages of leaf development, the majority of AS transcripts were enriched in vegetative organs. This unexpected observation may result from active transcription of flavonoid biosynthesis pathway genes and the rapid accumulation of flavonoid in leaf; this observation is in agreement with previous studies that found that the frequency of AS occurrence closely associated with the degree of tissue development and location during plant development [25, 46, 47].

**Fig. 6:**
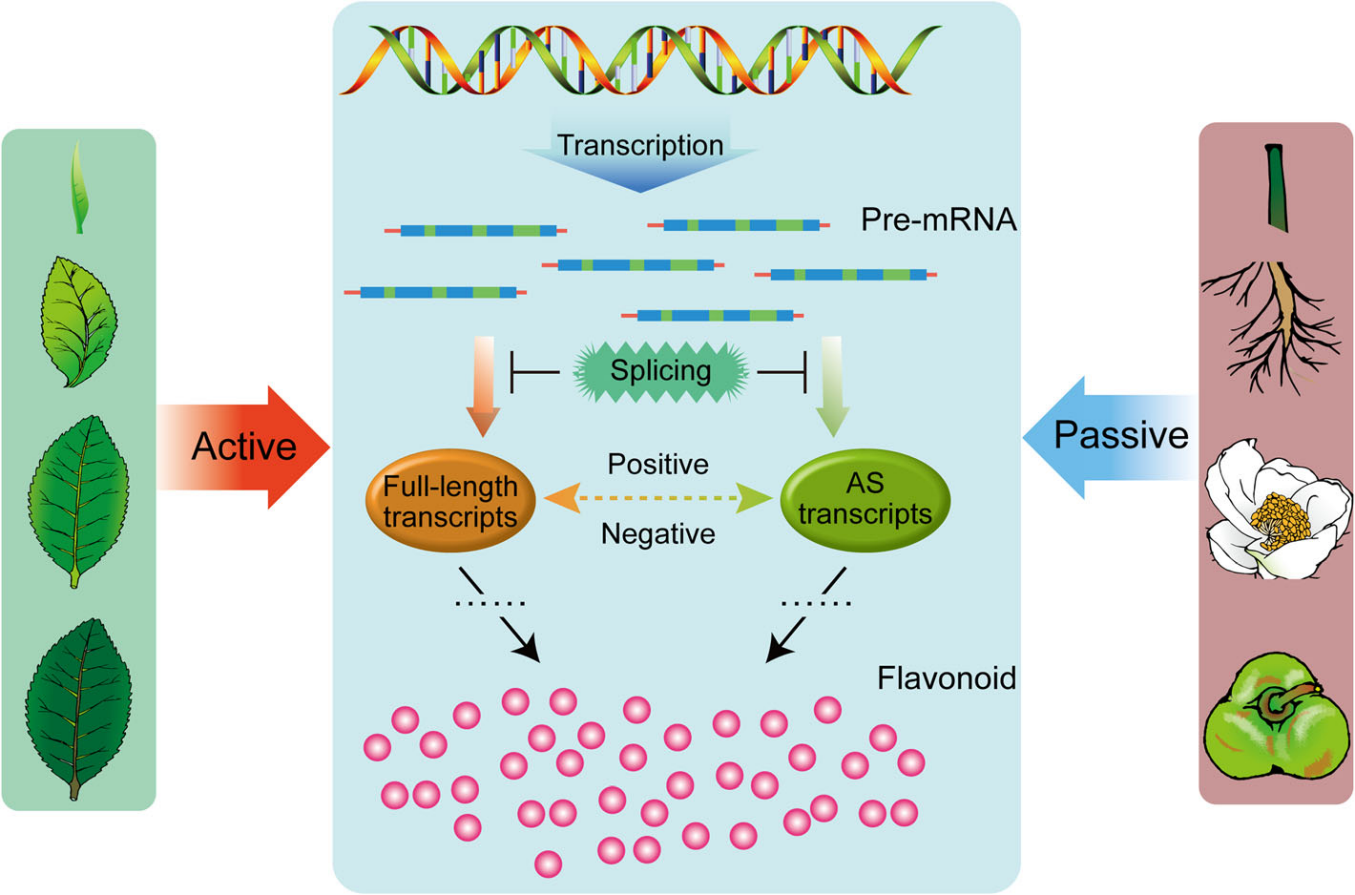
Hypothetical model for functions of alternative splicing in tea plant. The biosynthesis of flavonoid is active in vegetative tissues (bud, young leaf, summer mature leaf and winter old leaf) but passive in other tissues (stem, root, flower and fruit). As details of the processes of flavonoid biosynthesis, the full-length and AS transcripts synergistically functions and positive and negative relationship are exhibited between them at transcription level

Recent studies on protein LSm5 and SKIP indicated that the presence of AS transcripts generally decrease the levels of the corresponding full-length transcripts [48, 49]. Further expression analysis of flavonoid-derived AS genes showed they exhibit a pattern with low expression levels of AS transcripts relative to those of the full-length transcripts (Fig. 5a), which is agreement with previous reports [50, 51]. However, in contrast, the AS transcripts of *CsPAL* (TEA003146.1) and *CsMYB* (TEA012130.1) were predominant and displayed the opposite expression pattern relative to full-length transcripts in different tissues (Fig. 5b); this implies that the two AS transcripts likely serve as largely regulated or functional transcripts instead of the corresponding full-length transcripts. As a precedent for this idea, the circadian clock gene AtZ*TL* was shown to have its AS transcripts translated into authentic functional protein instead of its full-length transcripts [52, 53]. In addition, this hypothesis is supported by correlation analysis with the accumulation of catechins, which showed the two AS transcripts of *CsMYBβ* and *CsPALβ* are significantly correlated with epicatechin-3-gallate (ECG) levels (Table 2). Collectively, our data propose and support the hypothesis and that achievement of gene function not only depends on conventional full-length transcripts, but also on the extraordinary AS transcripts, which may act as major mediators at the post-transcriptional level during plant development (Fig. 6). The key determinants are the specific functions that the predominant AS transcripts confer and the dynamic equilibrium of abundance between the full-length and AS transcripts.

## Conclusions

The genome-scaled approach to investigation of tea plant development is surprisingly informative in uncovering transcriptional diversity mediated by alternative splicing. Based on the results of this study, we propose that the tissue-specific gene function and flavonoid biosynthesis could potentially be affected by an alternative splicing regulatory mechanism. This study provides the insight into the AS events underlying tea plant’s uniquely different developmental process and highlights the important contribution and efficacy of alternative splicing regulatory function to biosynthesis of flavonoids.

## Methods

### Plant resources

Tea plants (*C. sinensis cv. Shuchazao*) were grown in the 916 Tea Plantation in Shucheng County (latitude 31.3 N, longitude 117.2E above sea level), Anhui Province, China under natural conditions. Eight samples of different tissues were collected from exactly the same tea plant. The tissues sampled were taken as follows: bud, young leaf, summer mature leaf, winter old leaf, stems, flowers, fruits and roots. Bud, young leaf, summer mature leaf and stems were collected on June 15,2015; Winter old leaf were collected on November 13, 2015; Flowers, fruits and roots were collected on October 12, 2015.

At the same periods in 2016, the four tissues (bud, young leaf, root and flower) were collected again from exactly the same tea plant. All samples in this study were performed with three biological replicates and all collected samples were immediately frozen in liquid nitrogen and stored at − 80 °C until use.

### Transcriptomic data

Single molecule real-time DNA sequencing data was generated from individual pools of eight tea plant tissues (bud, young leaf, mature leaf in summer, old leaf in winter, stem, root, flower and seed) using the PacBio RS II platform (Pacific Biosciences of California, USA; Accession, SRR5460108) and data were reported in our previous study [18]. Additional RNA sequencing data was generated from 90-bp paired-end RNA-Seq reads from the aforementioned eight tissue samples using the Illumina Hiseq 2000 platform (Illumina, USA; Accession, PRJNA274203).

### Alignment of reads to the reference tea plant genome and gene expression estimation

We used Bowtie v2.0.6 to build an index of the tea plant (*Camellia sinensis*) reference genome [54]. The clean RNA sequence data from each sample were mapped to the tea plant (*Camellia sinensis*) genome using Tophat (v.2.0.11) [55].The maximum alignment number for each read was set to 2, and the inner distance between mating pairs was set to 230 bp. Mapped reads were assembled with Cufflinks v.2.1.1 to generate a genome-based Cufflinks assembly [56]. The rest of the parameters were set as default. We calculated reads per kilobase of exon model per million mapped fragments (FPKM) of genes based on gene length and the number of fragments mapped to corresponding genes [56].

### Identification and characterization of alternative splicing

The AS events were extracted from assembly files of different tissues with the software tool AStalavista and visualized by IGV browser [57, 58]. To minimize the mismatch rate, we required that the overhang size had to be greater than 25 bp with at least two reads spanning the junctions. The alternative splicing events were classified by using the gtf files assembled from the Illumina RNA-seq and SMRT sequencing data based on the tool AStalavista [57]. Four major types of AS events, namely IR (AS code: 1^2-,0), ES (AS code:1–2^,0), AA (AS code: 1-,2-), and AD (AS code: 1^,2^), were identified from the output files and counted, respectively.

### Co-expression network analysis

WGCNA (v1.29) package in R [26] was employed to construct co-expression networks. All AS genes were used for WGCNA unsigned co-expression network analysis; the corresponding FPKM of each gene was imported into WGCNA. The automatic network construction function block wise Modules values were set as default to construct the modules, except that the power was set to 18, minModuleSize to 30, and merge CutHeight to 0.35. The eigengene value was calculated for each module and used to test the association with each tissue type.

### Experimental validation of alternative splicing

Total RNA was isolated from the different tissues of tea plant as described above for RNA-seq. First-strand cDNA was synthesized from total RNA using a PrimeScript RT Reagent Kit (Takara, Japan) referred to the manufacturer’s instructions. Total AS events were validated by RT-PCR according to a reported protocol using P1-P42 primers that were designed based on each AS event.All specific primers are listed in Additional file 1: Table S5.

### Gene structures and phylogenetic analysis

The alternative splicing isoforms were viewed using the IGV software and were corrected with the SMRT sequencing reads. Gene structures were analyzed with online website Gene Structure Display Server (GSDS 2.0, http://gsds.cbi.pku.edu.cn/index.php). The phylogenetic relationships of candidate MYBs from *Camellia sinensis* and other species were analyzed by MEGA 6.0using NJ (Neighbor Joining) method with the following parameters:bootstrap method (1000 replicates), Poisson model, Uniform rates, Complete deletion [32].

### Extraction and HPLC analysis of catechins

Catechins were extracted from the eight tissues according to the reported method [59, 60]. 0.1 g of the freeze-dried tissues were ground in liquid nitrogen with a mortar and pestle and extracted with 3 mL 80% methanol in an ultrasonic sonicator for 10 min at 4 °C. After centrifugation at6,000 rpm for 10 min, the residues were re-extracted twice as described above. The supernatants were combined and diluted with 80% methanol to a volume of 10 mL. The obtained supernatants were filtered through a 0.22 μm organic membrane before HPLC analysis. Catechins contents were determined using Waters 2695 HPLC system with a reverse phase C18 column (Phenomenex 250 mm × 4.6 mm, 5 μm). The eluent was composed of 0.17% (*v*/v) acetic acid (A) in water and 100% acetonitrile (B) at 1 mL/min and the effluent was monitored at 278 nm [60]. The filtered sample (10 μL) was injected into the HPLC system for analysis. Samples from the eight tissues were analyzed in triplicate.

## Additional file

Additional file 1: **Figure S1.** Hierarchical cluster tree showing co-expression modules identified by WGCNA. **Figure S2.** The proposed pathway related to flavonoid biosynthesis. Black and red numbers in brackets following each gene indicate the number of expressed full-length and AS genes identified in eight tissues, respectively. **Figure S3.** Alternative spliced isoforms of theanine-related genes in tea plant. The orange solid dot indicate the targeted AS transcripts identified in specific tissues. **Figure S4.** Alternatively spliced isoforms of caffeine-related genes in tea plant. **Figure S5.** An unrooted neighbor joining phylogenetic tree constructed from 12 amino acid sequences of *MYBs* identified in *C. sinensis *and other species. The *CsMYB* genes are highlighted by a solid purple circle. The bold font indicates the *CsMYBs* which were identified as alternatively spliced genes. **Figure S6. **Validation of AS isoforms using independent samples by RT-PCR. The AS isoforms were verified by RT-PCR in four tissues (bud, young leaf, root and flower) and each tissue was performed with three biological replicates. Lane 1, 500 bp DNA maker. Lane 2, 6, 10 and 14, full-length transcripts of corresponding genes. Lane 3, 4 and 5, AS isforms in bud. Lane 7, 8 and 9, AS isoforms in young leaf. Lane 11, 12 and 13, AS isoforms in root. Lane 15, 16 and 17, AS isoforms in flower. **Table S1.** Statistics of different AS events obtained from the Illumina libraries generated in this study. **Table S2.** Statistic analysis of characterization of identified AS genes in different tissues. **Table S3.** Statistics of different AS events obtained from the specific modules based on WGCNA analysis. **Table S4.** Statistical analysis of characterized AS genes identified in different tissues. The solid dot indicated the targeted AS transcripts identified in specific tissues. **Table S5.** Primers used in this study (application details of primers are described in the materials and method. (PDF 1036 kb)

## Abbreviations

AA: Alternative acceptor sites
AD: Alternative donor sites
AS: Alternative splicing
B: Bud
ECG: Epicatechin-3-gallate
EGC: Epigallocatechin
ES: Exon skipping
FL: Flower
FR: Fruit
HPLC: High performance liquid chromatography
IR: Intron retention
KEGG: Kyoto Encyclopedia of Genes and Genomes
MXE: Mutually exclusive exon
NMD: Nonsense-mediated decay
Pre-mRNA: Precursor-mRNA
PTC: Premature stop codon
R: Root
RNA-Seq: RNA sequencing
RT-PCR: Reverse transcriptase polymerase chain reaction
S: Stem
SML: Summer mature leaf
SR: Serine/arginine-rich
WGCNA: Weighted Gene Co-Expression Network Analysis
WOL: Winter old leaf
YL: Young leaf

## References

[1] Shi CY, Yang H, Wei CL, Yu O, Zhang ZZ, Jiang CJ, Sun J, Li YY, Chen Q, Xia T, Wan XC (2011). Deep sequencing of the Camellia sinensis transcriptome revealed candidate genes for major metabolic pathways of tea-specific compounds. BMC Genomics.

[2] Zaveri NT (2006). Green tea and its polyphenolic catechins: medicinal uses in cancer and noncancer applications. Life Sci.

[3] Reto M, Figueira ME, Filipe HM, Almeida CM (2007). Chemical composition of green tea (Camellia sinensis) infusions commercialized in Portugal. Plant Foods Hum Nutr.

[4] Li C-F, Zhu Y, Yu Y, Zhao Q-Y, Wang S-J, Wang X-C, Yao M-Z, Luo D, Li X, Chen L, Yang Y-J. Global transcriptome and gene regulation network for secondary metabolite biosynthesis of tea plant (Camellia sinensis). BMC Genomics. 2015;16:539–60. 10.1186/s12864-015-1773-0 PMC4518527 26220550

[5] Guo F, Guo Y, Wang P, Wang Y, Ni D (2017). Transcriptional profiling of catechins biosynthesis genes during tea plant leaf development. Planta.

[6] Staiger D, Brown JW (2013). Alternative splicing at the intersection of biological timing, development, and stress responses. Plant Cell.

[7] Reddy AS, Marquez Y, Kalyna M, Barta A (2013). Complexity of the alternative splicing landscape in plants. Plant Cell.

[8] Kelemen O, Convertini P, Zhang Z, Wen Y, Shen M, Falaleeva M, Stamm S (2013). Function of alternative splicing. Gene.

[9] Filichkin S, Priest HD, Megraw M, Mockler TC (2015). Alternative splicing in plants: directing traffic at the crossroads of adaptation and environmental stress. Curr Opin Plant Biol.

[10] Rosloski SM, Singh A, Jali SS, Balasubramanian S, Weigel D, Grbic V (2013). Functional analysis of splice variant expression of MADS AFFECTING FLOWERING 2 of Arabidopsis thaliana. Plant Mol Biol.

[11] Wang C, Tian Q, Hou Z, Mucha M, Aukerman M, Olsen OA (2007). The Arabidopsis thaliana AT PRP39-1 gene, encoding a tetratricopeptide repeat protein with similarity to the yeast pre-mRNA processing protein PRP39, affects flowering time. Plant Cell Rep.

[12] Keller M, Hu Y, Mesihovic A, Fragkostefanakis S, Schleiff E, Simm S (2017). Alternative splicing in tomato pollen in response to heat stress. DNA Res.

[13] Seo PJ, Park MJ, Lim MH, Kim SG, Lee M, Baldwin IT, Park CM (2012). A self-regulatory circuit of CIRCADIAN CLOCK-ASSOCIATED1 underlies the circadian clock regulation of temperature responses in Arabidopsis. Plant Cell.

[14] James AB, Syed NH, Bordage S, Marshall J, Nimmo GA, Jenkins GI, Herzyk P, Brown JWS, Nimmo HG (2012). Alternative splicing mediates responses of the Arabidopsis circadian clock to temperature changes. Plant Cell.

[15] Iida K, Seki M, Sakurai T, Satou M, Akiyama K, Toyoda T, Konagaya A, Shinozaki K (2004). Genome-wide analysis of alternative pre-mRNA splicing in Arabidopsis thaliana based on full-length cDNA sequences. Nucleic Acids Res.

[16] Blencowe BJ (2006). Alternative splicing: new insights from global analyses. Cell.

[17] Kriechbaumer V, Wang P, Hawes C, Abell BM (2012). Alternative splicing of the auxin biosynthesis gene YUCCA4 determines its subcellular compartmentation. Plant J.

[18] Xu Q, Zhu J, Zhao S, Hou Y, Li F, Tai Y, Wan X, Wei C (2017). Transcriptome profiling using single-molecule direct RNA sequencing approach for in-depth understanding of genes in secondary metabolism pathways of Camellia sinensis. Front Plant Sci.

[19] Yuan Y, Chung JD, Fu X, Johnson VE, Ranjan P, Booth SL, Harding SA, Tsai CJ (2009). Alternative splicing and gene duplication differentially shaped the regulation of isochorismate synthase in Populus and Arabidopsis. Proc Natl Acad Sci U S A.

[20] Gorlach J, Raesecke HR, Abel G, Wehrli R, Amrhein N, Schmid J (1995). Organ-specific differences in the ratio of alternatively spliced chorismate synthase (LeCS2) transcripts in tomato. Plant J.

[21] Huang W, Sun W, Lv H, Luo M, Zeng S, Pattanaik S, Yuan L, Wang Y (2013). A R2R3-MYB transcription factor from Epimedium sagittatum regulates the flavonoid biosynthetic pathway. PLoS One.

[22] Grotewold E, Athma P, Peterson T (1991). Alternatively spliced products of the maize P gene encode proteins with homology to the DNA-binding domain of myb-like transcription factors. Proc Natl Acad Sci U S A.

[23] Filichkin SA, Priest HD, Givan SA, Shen R, Bryant DW, Fox SE, Wong WK, Mockler TC (2010). Genome-wide mapping of alternative splicing in Arabidopsis thaliana. Genome Res.

[24] Walters B, Lum G, Sablok G, Min XJ (2013). Genome-wide landscape of alternative splicing events in Brachypodium distachyon. DNA Res.

[25] Shen Y, Zhou Z, Wang Z, Li W, Fang C, Wu M, Ma Y, Liu T, Kong LA, Peng DL, Tian Z (2014). Global dissection of alternative splicing in paleopolyploid soybean. Plant Cell.

[26] Langfelder P, Horvath S (2008). WGCNA: an R package for weighted correlation network analysis. BMC Bioinformatics.

[27] Sanchez P, Nehlin L, Greb T (2012). From thin to thick: major transitions during stem development. Trends Plant Sci.

[28] Didonato RJ, Arbuckle E, Buker S, Sheets J, Tobar J, Totong R, Grisafi P, Fink GR, Celenza JL (2004). Arabidopsis ALF4 encodes a nuclear-localized protein required for lateral root formation. Plant J.

[29] Krizek Beth A., Fletcher Jennifer C. (2005). Molecular mechanisms of flower development: an armchair guide. Nature Reviews Genetics.

[30] Wei C, Yang H, Wang S, Zhao J, Liu C, Gao L, et al. Draft genome sequence of Camellia sinensis var. sinensis provides insights into the evolution of the tea genome and tea quality. Proc Natl Acad Sci U S A. 2018;115(18): E4151–E4158. 10.1073/pnas.1719622115 PMC5939082 29678829

[31] Willson KC, Clifford MN. Tea: cultivation to consumption Berlin: Springer Science & Business Media, Chapman & Hall; 1992. p. 175–182.

[32] Liu J, Osbourn A, Ma P (2015). MYB transcription factors as regulators of Phenylpropanoid metabolism in plants. Mol Plant.

[33] Hori K, Watanabe Y (2007). Context analysis of termination codons in mRNA that are recognized by plant NMD. Plant Cell Physiol.

[34] Li W, Xiang F, Zhong M, Zhou L, Liu H, Li S, Wang X (2017). Transcriptome and metabolite analysis identifies nitrogen utilization genes in tea plant (Camellia sinensis). Sci Rep.

[35] Iida K, Go M (2006). Survey of conserved alternative splicing events of mRNAs encoding SR proteins in land plants. Mol Biol Evol.

[36] Kalyna M, Lopato S, Voronin V, Barta A (2006). Evolutionary conservation and regulation of particular alternative splicing events in plant SR proteins. Nucleic Acids Res.

[37] Jiao Y, Tausta SL, Gandotra N, Sun N, Liu T, Clay NK, Ceserani T, Chen M, Ma L, Holford M, Zhang HY, Zhao H, Deng XW, Nelson T (2009). A transcriptome atlas of rice cell types uncovers cellular, functional and developmental hierarchies. Nat Genet.

[38] Wang L, Cao C, Ma Q, Zeng Q, Wang H, Cheng Z, Zhu G, Qi J, Ma H, Nian H (2014). RNA-seq analyses of multiple meristems of soybean: novel and alternative transcripts, evolutionary and functional implications. BMC Plant Biol.

[39] Honys D, Twell D (2003). Comparative analysis of the Arabidopsis pollen transcriptome. Plant Physiol.

[40] Li P, Ponnala L, Gandotra N, Wang L, Si Y, Tausta SL, Kebrom TH, Provart N, Patel R, Myers CR, Reidel EJ, Turgeon R, Liu P, Sun Q, Nelson T, Brutnell TP (2010). The developmental dynamics of the maize leaf transcriptome. Nat Genet.

[41] Wang B, Tseng E, Regulski M, Clark TA, Hon T, Jiao Y, Lu Z, Olson A, Stein JC, Ware D (2016). Unveiling the complexity of the maize transcriptome by single-molecule long-read sequencing. Nat Commun.

[42] Li Q, Li J, Liu S, Huang J, Lin H, Wang K, Cheng X, Liu Z (2015). A comparative proteomic analysis of the buds and the young expanding leaves of the tea plant (Camellia sinensis L.). Int J Mol Sci.

[43] Sun Y, Flannigan BA, Madison JT, Setter TL (1997). Alternative splicing of cyclin transcripts in maize endosperm. Gene.

[44] Koes RE, Quattrocchio F, Mol JNM (1994). The flavonoid biosynthetic pathway in plants: function and evolution. Bioessays.

[45] Hichri I, Barrieu F, Bogs J, Kappel C, Delrot S, Lauvergeat V (2011). Recent advances in the transcriptional regulation of the flavonoid biosynthetic pathway. J Exp Bot.

[46] Wang T, Wang H, Cai D, Gao Y, Zhang H, Wang Y, Lin C, MA L, Gu L (2017). Comprehensive profiling of rhizome-associated alternative splicing and alternative polyadenylation in moso bamboo (Phyllostachys edulis). Plant J.

[47] SUN Y, XIAO H (2015). Identification of alternative splicing events by RNA sequencing in early growth tomato fruits. BMC Genomics.

[48] Cui P, Zhang S, Ding F, Ali S, Xiong L (2014). Dynamic regulation of genome-wide pre-mRNA splicing and stress tolerance by the Sm-like protein LSm5 in Arabidopsis. Genome Biol.

[49] Feng J, Li J, Gao Z, Lu Y, Yu J, Zheng Q, Yan S, Zhang W, He H, Ma L, Zhu Z (2015). SKIP confers osmotic tolerance during salt stress by controlling alternative gene splicing in Arabidopsis. Mol Plant.

[50] Shen Y, Wu X, Liu D, Song S, Liu D, Wang H (2016). Cold-dependent alternative splicing of a Jumonji C domain-containing gene MtJMJC5 in Medicago truncatula. Biochem Biophys Res Commun.

[51] Park SY, Grabau E (2016). Differential isoform expression and protein localization from alternatively spliced Apetala2 in peanut under drought stress. J Plant Physiol.

[52] Mas P, Kim WY, Somers DE, Kay SA (2003). Targeted degradation of TOC1 by ZTL modulates circadian function in Arabidopsis thaliana. Nature.

[53] Somers DE, Schultz TF, Milnamow M, Kay SA (2000). ZEITLUPE encodes a novel clock-associated PAS protein from Arabidopsis. Cell.

[54] Langmead B, Salzberg SL (2012). Fast gapped-read alignment with bowtie 2. Nat Methods.

[55] Trapnell C, Pachter L, Salzberg SL (2009). TopHat: discovering splice junctions with RNA-Seq. Bioinformatics.

[56] Trapnell C, Roberts A, Goff L, Pertea G, Kim D, Kelley DR, Pimentel H, Salzberg SL, Rinn JL, Pachter L (2012). Differential gene and transcript expression analysis of RNA-seq experiments with TopHat and cufflinks. Nat Protoc.

[57] Foissac S, Sammeth M (2007). ASTALAVISTA: dynamic and flexible analysis of alternative splicing events in custom gene datasets. Nucleic Acids Res.

[58] Thorvaldsdottir H, Robinson JT, Mesirov JP (2013). Integrative genomics viewer (IGV): high-performance genomics data visualization and exploration. Brief Bioinform.

[59] Shan Y, Wei-Wei LI, Wang YS, Liu YJ, Wang HX, Wang XF, Zhong-Wei LU, Tian YW, Gao LP, Xia T. Catechins synthesis and accumulation in tea seedlings at different development stages. J Anhui Agric Univ. 2011;38:4.

[60] Tai Y, Wei C, Yang H, Zhang L, Chen Q, Deng W, Wei S, Zhang J, Fang C, Ho C, Wan X. Transcriptomic and phytochemical analysis of the biosynthesis of characteristic constituents in tea (Camellia sinensis) compared with oil tea (Camellia oleifera). BMC Plant Biol. 2015;15:1–13. 10.1186/s12870-015-0574-6 PMC4527363 26245644

