# Supplementary material for: Global dissection of alternative splicing uncovers transcriptional diversity in tissues and associates with the flavonoid pathway in tea plant (Camellia sinensis)
Source: BMC Plant Biol. 2018 Nov 6;18:266. doi: 10.1186/s12870-018-1497-9 (PMC6219262; doi:10.1186/s12870-018-1497-9)
Supplement: Supplementary file 1 — Figure S1. Hierarchical cluster tree showing co-expression modules identified by WGCNA. Figure S2. The proposed pathway related to flavonoid biosynthesis. Black and red numbers in brackets following each gene indicate the number of expressed full-length and AS genes identified in eight tissues, respectively. Figure S3. Alternative spliced isoforms of theanine-related genes in tea plant. The orange solid dot indicate the targeted AS transcripts identified in specific tissues. Figure S4. Alternatively spliced isoforms of caffeine-related genes in tea plant. Figure S5. An unrooted neighbor joining phylogenetic tree constructed from 12 amino acid sequences of MYBs identified in C. sinensis and other species. The CsMYB genes are highlighted by a solid purple circle. The bold font indicates the CsMYBs which were identified as alternatively spliced genes. Figure S6. Validation of AS isoforms using independent samples by RT-PCR. The AS isoforms were verified by RT-PCR in four tissues (bud, young leaf, root and flower) and each tissue was performed with three biological replicates. Lane 1, 500 bp DNA maker. Lane 2, 6, 10 and 14, full-length transcripts of corresponding genes. Lane 3, 4 and 5, AS isforms in bud. Lane 7, 8 and 9, AS isoforms in young leaf. Lane 11, 12 and 13, AS isoforms in root. Lane 15, 16 and 17, AS isoforms in flower. Table S1. Statistics of different AS events obtained from the Illumina libraries generated in this study. Table S2. Statistic analysis of characterization of identified AS genes in different tissues. Table S3. Statistics of different AS events obtained from the specific modules based on WGCNA analysis. Table S4. Statistical analysis of characterized AS genes identified in different tissues. The solid dot indicated the targeted AS transcripts identified in specific tissues. Table S5. Primers used in this study (application details of primers are described in the materials and method. (PDF 1036 kb) [file 12870_2018_1497_MOESM1_ESM.pdf]

Supplementary

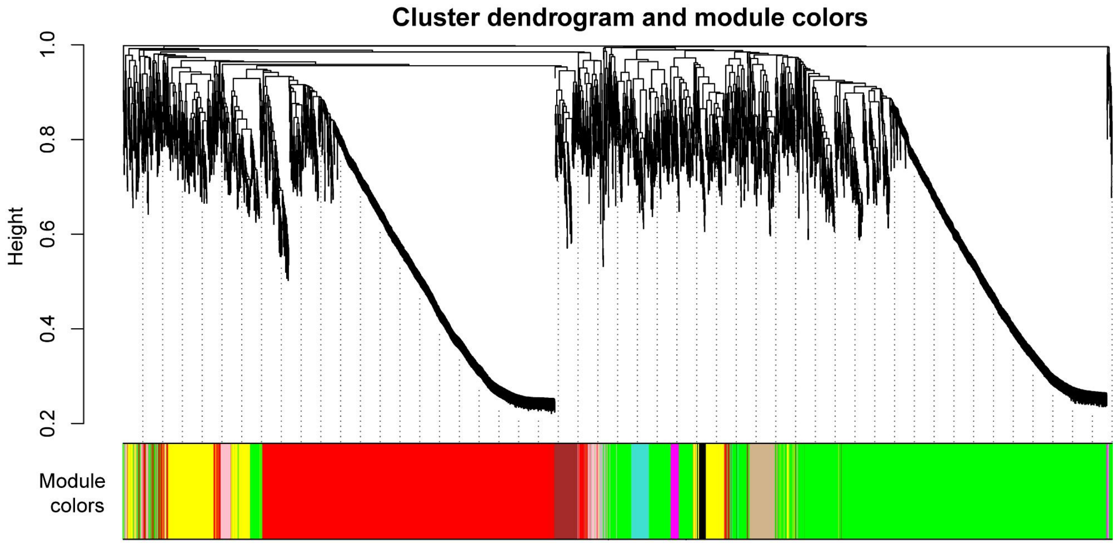

Supplementary Figure S1. Hierarchical cluster tree showing co-expression modules identified by WGCNA. Each leaf in the tree represents one gene. The major tree branches constitute the 13 modules indicated by different colors.

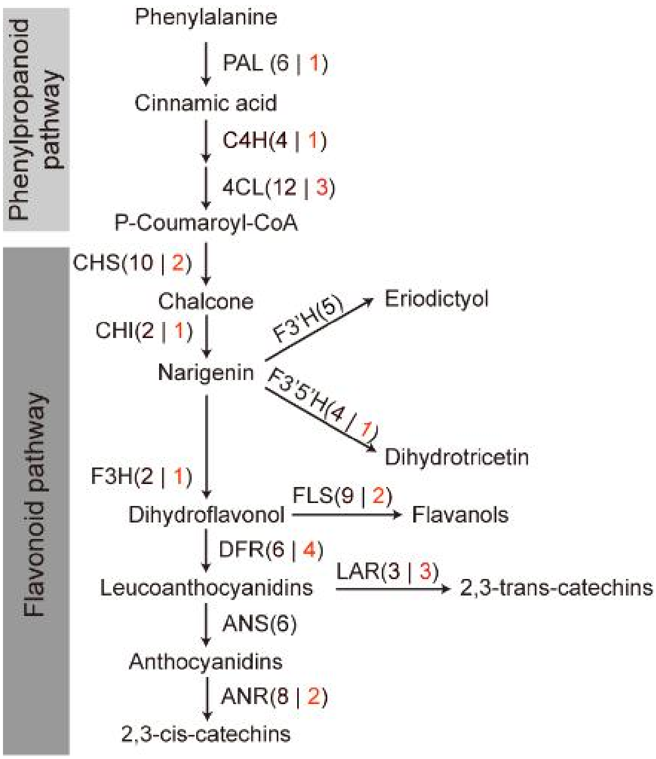

Supplementary Figure S2. The proposed pathway related to flavonoid biosynthesis. Black and red numbers in brackets following each gene indicate the number of expressed full-length and AS genes identified in eight tissues, respectively.

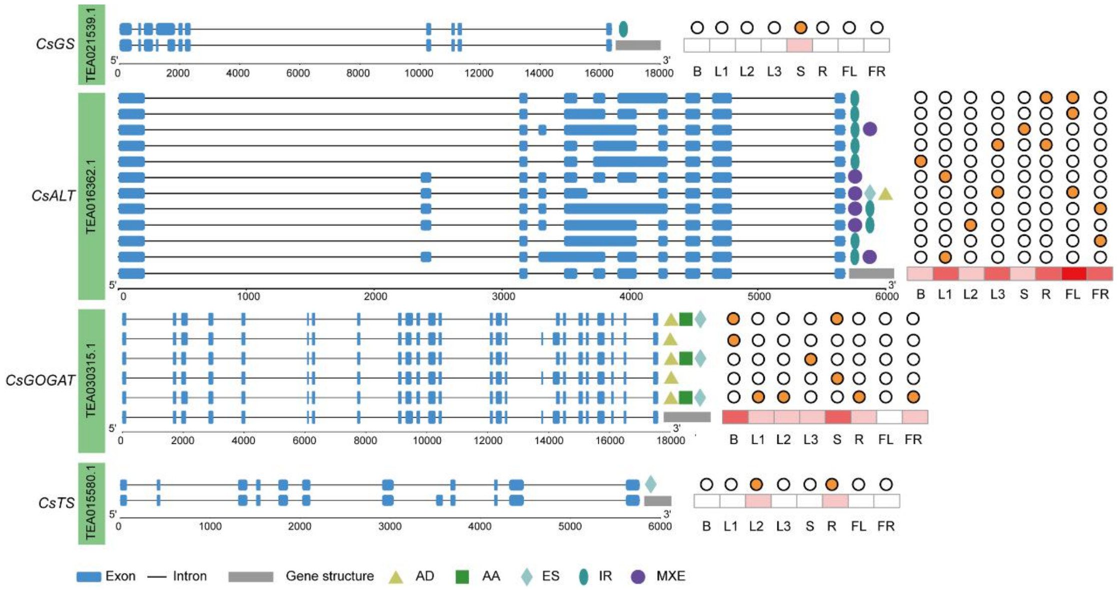

Supplementary Figure S3. Alternative spliced isoforms of theanine-related genes in tea plant. The orange solid dot indicate the targeted AS transcripts identified in specific tissues (B, bud; YL, young leaf; SML, summer mature leaf; WOL, winter old leaf; S, stem; R, root; FL, flower; FR, fruit).

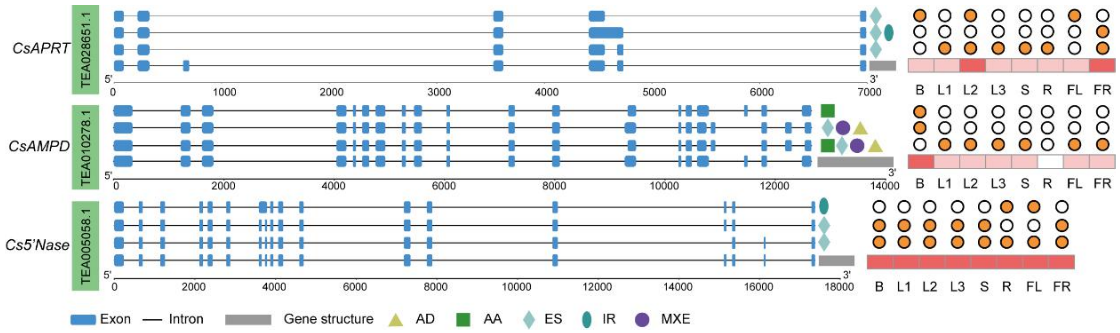

Supplementary Figure S4. Alternatively spliced isoforms of caffeine-related genes in tea plant. The orange solid dot indicates the targeted AS transcripts identified in specific tissues (B, bud; YL, young leaf; SML, summer mature leaf; WOL, winter old leaf; S, stem; R, root; FL, flower; FR, fruit).

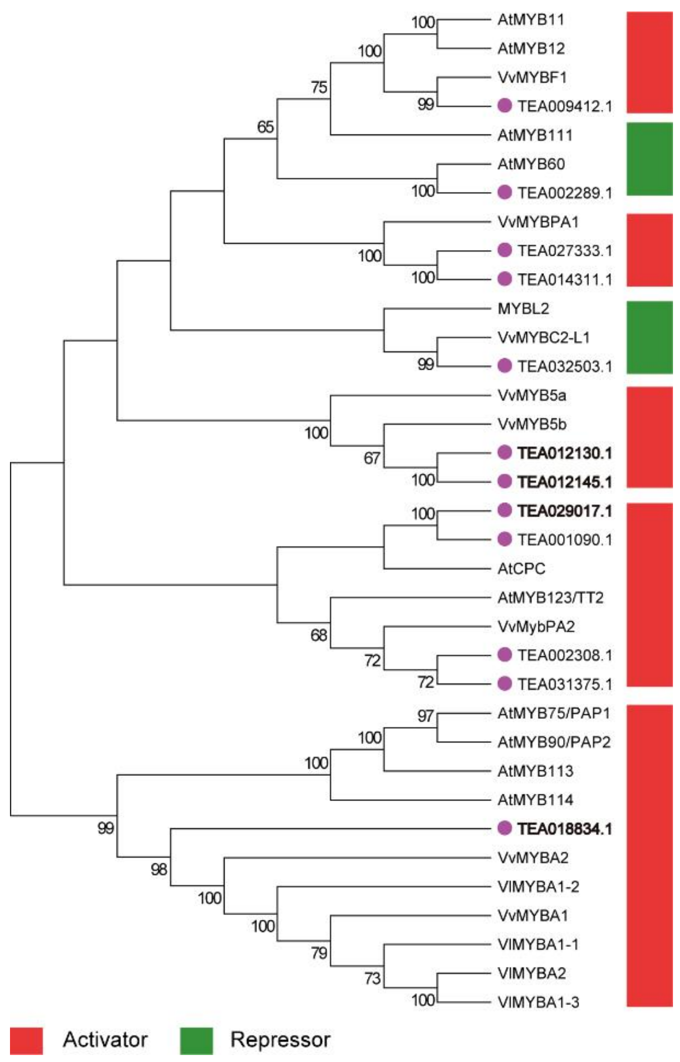

Supplementary Figure S5. An unrooted neighbor joining phylogenetic tree constructed from 12 amino acid sequences of MYBs identified in *C. sinensis* and other species. A bootstrap test is set as 1000 to validate the confidence of the classifications. The bootstrap values of confidence levels are shown as percentages at the branch nodes. The *CsMYB* genes are highlighted by a solid purple circle. The bold font indicates the *CsMYBs* which were identified as alternatively spliced genes. AtMYB11 (NP_191820), AtMYB12 (NP_182268), AtMYB60 (NP_172358), AtMYB111 (NP_199744), AtMYB123 (NP_198405), AtCPC (NM_130205), AtMYB75 (NP_176057), AtMYB90 (NP_176813), AtMYB113 (NP_176811), AtMYB114 (NP_176812), VvMYBF1 (GQ423422), VvMYBPA1 (AM259485), VvMYBC2-L1 (JX050227), VvMYB5a (AY555190), VvMYB5b (AY899404), VvMYBPA2 (EU919682), VvMYBA1 (AB097923), VvMYBA2 (AB097924), VlMYBA1-1 (AB073010), VlMYBA1-2 (AB073012), VlMYBA2 (AB073013), VlMYBA1-3 (AB427165).

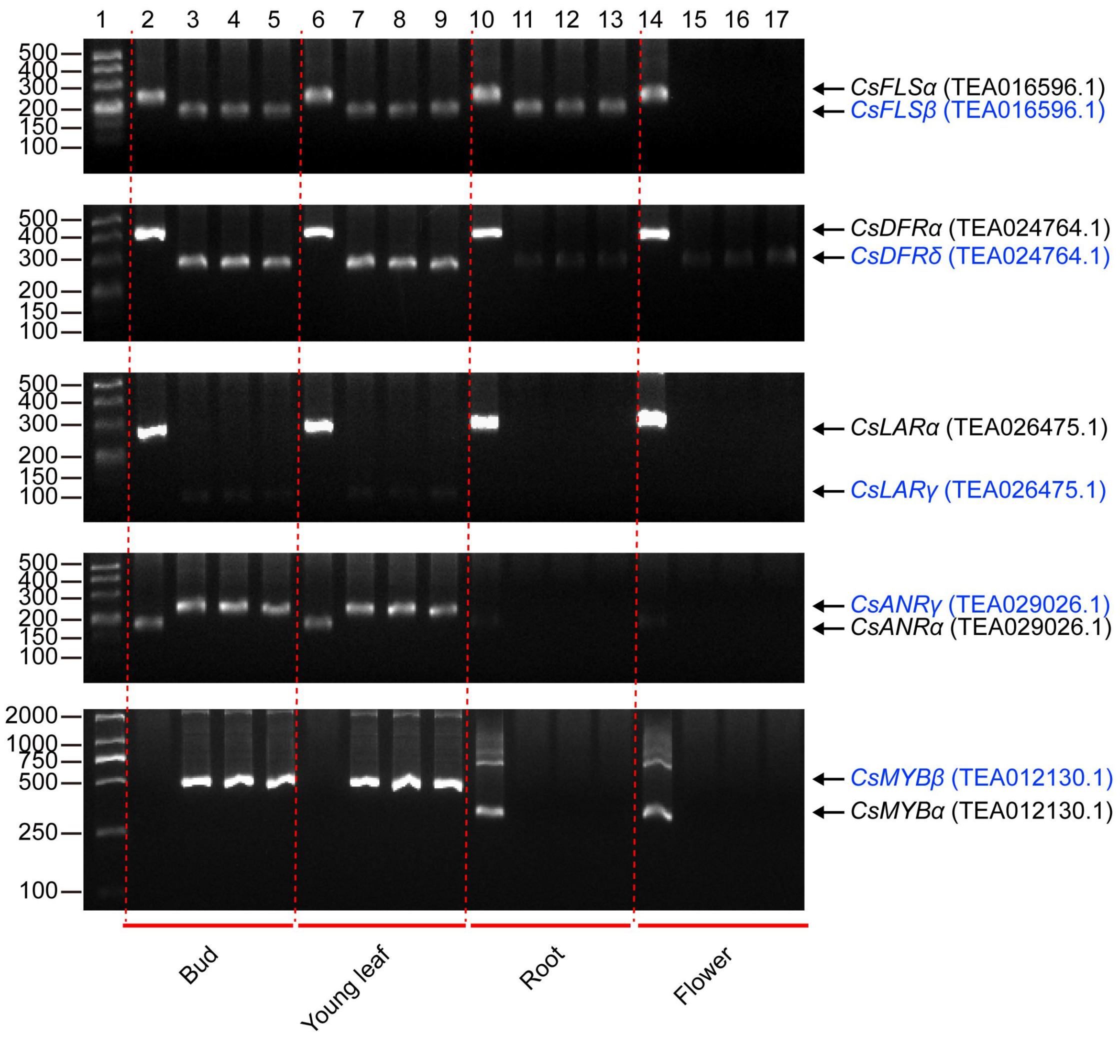

Supplementary Figure S6. Validation of AS isoforms using independent samples by RT-PCR. The AS isoforms were verified by RT-PCR in four tissues (bud, young leaf, root and flower) and each tissue was performed with three biological replicates. Lane 1, 500bp DNA maker. Lane 2, 6, 10 and 14, full-length transcripts of corresponding genes. Lane 3, 4 and 5, AS isforms in bud. Lane 7, 8 and 9, AS isoforms in young leaf. Lane 11, 12 and 13, AS isoforms in root. Lane 15, 16 and 17, AS isoforms in flower. β isoforms marked with blue represent the AS isoforms, α.isoforms marked with black represent the full-length transcripts.

Table. S1 Statistics of different AS events obtained from the Illumina libraries generated in this study

	Illumina															
	Bud		Young leaf		Summer mature leaf		Winter old leaf		Stem		Root		Flower		Fruit	
	Number	Percentage	Number	Percentage	Number	Percentage	Number	Percentage	Number	Percentage	Number	Percentage	Number	Percentage	Number	Percentage
Alternative donor site	3911	16.17%	3831	16.01%	3961	16.09%	4155	16.07%	4078	15.98%	3659	16.12%	3723	16.20%	3693	16.02%
Alternative acceptor site	4261	17.62%	4226	17.66%	4289	17.43%	4563	17.65%	4568	17.90%	3940	17.36%	4068	17.70%	4115	17.85%
Intron retention	4753	19.65%	4727	19.76%	4807	19.53%	5314	20.55%	5111	20.03%	4470	19.69%	4446	19.34%	4660	20.22%
Exon skipping	4055	16.77%	4008	16.75%	4177	16.97%	4417	17.08%	4205	16.47%	3913	17.24%	3939	17.14%	3769	16.35%
Others	7199	29.79%	7127	29.82%	7371	29.98%	7402	28.65%	7554	29.62%	6711	29.59%	6801	29.62%	6808	29.56%
Total events	24179	100.00%	23919	100.00%	24605	100.00%	25851	100.00%	25516	100.00%	22693	100.00%	22977	100.00%	23045	100.00%

Table. S2 Statistic analysis of characterization of identified AS genes in different tissues

	AS types
Serine/arginine-rich protein	
TEA002926.1	AA
TEA029911.1	ES
TEA027329.1	IR
TEA008450.1	ES
TEA025233.1	ES
TEA004460.1	AA,AD
TEA008159.1	ES
TEA023301.1	ES
U2af-like protein	
TEA007383.1	ES
TEA027719.1	ES
TEA025294.1	AD
TEA031074.1	IR
TEA002124.1	AA
3B-like protein	
TEA033372.1	ES
TEA002823.1	AA
TEA023785.1	IR

AA (Alternative Acceptor site), IR (Intron Retention), AD (Alternative Donor site), ES (Exon Skipping).

Table. S3 Statistics of different AS events obtained from the specific modules based on WGCNA analysis

	Illumina											
	Black		Blue		Pink		Green		Red		Brown	
	Number	Percentage	Number	Percentage	Number	Percentage	Number	Percentage	Number	Percentage	Number	Percentage
Alternative donor site	339	16.22%	739	15.79%	296	17.98%	429	16.50%	420	17.07%	511	15.32%
Alternative acceptor site	375	17.94%	813	17.37%	300	18.22%	437	16.81%	460	18.69%	568	17.03%
Intron retention	352	16.84%	878	18.76%	313	19.01%	577	22.20%	445	18.08%	705	21.13%
Exon skipping	370	17.70%	827	17.67%	245	14.88%	412	15.85%	396	16.09%	550	16.49%
Others	654	31.30%	1421	30.41%	492	29.91%	744	28.64%	739	30.07%	1001	30.03%
Total events	2090	100.00%	4678	100.00%	1646	100.00%	2599	100.00%	2460	100.00%	3335	100.00%

Table. S4 Statistical analysis of characterized AS genes identified in different tissues

	Bud	Young leaf	Summer mature leaf	Winter old leaf	Stem	Root	Flower	Fruit	AS types	RNA-Seq technique
PAL										
TEA003146.1	●								AA	
4CL										Illumina
TEA031662.1	●	●	●						IR	
				●					IR,AA	

TEA018046.1	•	•	•	•			•	•	ES
TEA010673.1		•							IR
CHI									
TEA014872.1						•			ES
F3H									
TEA033515.1			•	•					AD
			•		•		•	•	IR,AD
FLS									
TEA016596.1	•	•				•			IR
TEA010325.1			•						AD
DFR									
TEA024764.1	•								ES
						•			ES
				•					ES,ME
TEA024761.1						•			ME,ES
TEA023789.1					•				IR
TEA010593.1					•				IR
LAR									
TEA026475.1	•	•			•				ES
		•							AD
TEA021539.1	•								AA
		•							ES
				•					IR
ANR									

TEA022952.1	●		●		●			IR	
TEA029026.1			●	●				IR	
				●				AD,IR	
MYB									
TEA012130.1	●	●						IR	
TEA029017.1	●	●	●					AA	
TEA018834.1							●	IR	
TEA012145.1						●		IR	
C4H									
TEA014865.1								ES	
4CL									
TEA018046.1								ES	
CHS									
TEA023332.1								AD	
TEA023340.1								IR	SMRT
F35H									
TEA013309.1								AD	
FLS									
TEA016596.1								IR	
LAR									
TEA027557.1								IR	
ANR									

TEA022952.1	IR<br>IR
MYB	
TEA012130.1	IR

AA (Alternative Acceptor site), IR (Intron Retention), AD (Alternative Donor site), ES (Exon Skipping). Illumina and SMRT indicates the AS genes are detected from Illumina paired-end RNA-seq data and Single molecule real-time DNA sequencing data, respectively. The solid dot indicated the targeted AS transcripts identified in specific tissues.

Table. S5 Primers used in this study (application details of primers are described in the materials and methods)

Primer name	Sequence(5'-3')	Target gene
P1-F	AAGAGCTCATTAGATTTTTGAATG	TEA003146.1α
P2-R	AAGCATGGAGTGATGTTGTG	
P3-F	CATGGCCAGCAGTGATTG	TEA003146.1β
P4-R	AAGCATGGAGTGATGTTGTG	
P5-F	AAGGCTATCAGGTTGCTCC	TEA031662.1α
P6-R	CATCTGGGTACGGAACCA	
P7-F	TAGTAGCGCAGGGTCATC	TEA031662.1β
P8-R	GATTTGTATTGCATTATTGCATAC	
P9-F	ATGGTCTGACAGAATCAACTG	TEA031662.1γ
P10-R	GATGCTAACCAAGATTGAATAG	
P1-F	TTCTAGAAGTACCGGTGATC	TEA016596.1α
P12-R	TGCTCTTAGACTCAGCATC	
P13-F	AACCATGGGATACCAGATG	TEA016596.1β

P14-R	CTTTCTTCCCTTCAACTTCC	
P15-F	ATGACTGGATGGATGTATTTTG	TEA024764.1α
P16-R	TTGAATCCTAGGTCCAACAG	
P17-F	ATGACTGGATGGGAAATGAAC	TEA024764.1β
P18-R	TTGAATCCTAGGTCCAACAG	
P19-F	ATCCTGAGATGTATTTTGTGG	TEA024764.1γ
P20-R	TTGAATCCTAGGTCCAACAG	
P21-F	AGCACTTCACCCAACGCT	TEA024764.1δ
P22-R	TTGAATCCTAGGTCCAACAG	
P23-F	GTTGATGGGTCTGATATTGG	TEA026475.1α
P24-R	CATCTGGATAGAGATTGCTC	
P25-F	ACTGTCAAAGTTGTGGACG	TEA026475.1β
P26-R	CAACTTCATTTGGACCATCAA	
P27-F	ACTGTCAAAGAGAATATCATAC	TEA026475.1γ
P28-R	TAACTTGACAACCCTTGATG	
P29-F	AGAGCTCTCAGTAATACATC	TEA029026.1α
P30-R	GAGAAGTCCAAGAGGATTC	
P31-F	ATTCATGATTTCTCTTGTTGTC	TEA029026.1β
P32-R	TTATAGCCAGACCCATCTTC	
P33-F	AGCTTCTTAGTAATACATCTG	TEA029026.1γ
P34-R	GAGAAGTCCAAGAGGATTC	
P35-F	CAGGTCTACAATCCGTATAG	TEA012130.1α
P36-R	CTCACCATGATAATACTGGC	
P37-F	TTCTACTACTTGTCAATTAGGG	TEA012130.1β

P38-R	CTCACCATGATAATACTGGC	
P39-F	ACGAACCGATAATGAGATTAAG	TEA029017.1α
P40-R	CACTTCCTTTGGTGCCTTC	
P41-F	ATTAGGGAACAGGAAAATAAATC	TEA029017.1β
P42-R	GACGTCAGAGTCTGCAGT	
